# Special Issue: Rare earth luminescent materials

**DOI:** 10.1038/s41377-022-00956-9

**Published:** 2022-09-02

**Authors:** Hongjie Zhang, Hong Zhang

**Affiliations:** 1 State Key Laboratory of Rare Earth Resource Utilization, Changchun Institute of Applied Chemistry, Chinese Academy of Sciences, Changchun, Jilin, 130022 China; 2 Department of Chemistry, Tsinghua University, Beijing, 100084 China; 3 Van’t Hoff Institute for Molecular Sciences, University of Amsterdam, P. O. Box 94157, 1090 GD Amsterdam, The Netherlands

**Keywords:** Optical materials and structures, Optics and photonics

## Abstract

This special issue covers a series of cutting-edge works on exploring novel rare earth luminescent materials and their applications in lighting, display, information storage, sensing, and bioimaging as well as therapy.

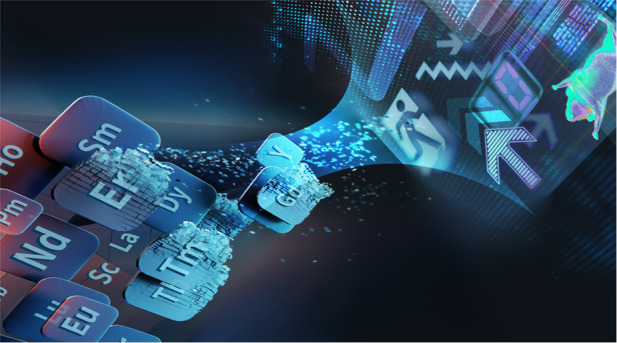

Trivalent rare earth ions (Ln^3+^) have the unique electronic configurations [Xe]4f^n^ (*n* = 0–14) and numerous energy levels, which endow rare earth luminescent materials with many fascinating optical properties over a broad spectral region ranging from the ultraviolet (UV) to the near infrared (NIR); these include tunable atomic-like excitation/emission spectra, large Stokes/anti-Stokes shifts, long luminescent lifetimes, and excellent photostability^1–4^. In the 1960s, thanks to breakthroughs in purification technology, rare earth luminescent materials were successfully applied in lamps and cathode-ray tubes as original phosphors^5^. Since then, with continuous and important progress arising from research on rare earth luminescent materials, applications have consistently been expanded. Today, rare earth luminescent materials are used in almost every aspect of photonics and optoelectronics, for example, in lighting^6,7^, displays^8,9^, sensing^10^, optical information storage^11^, energy conservation^12^, and biomedicine^13,14^. This booming momentum prompted us to edit this album and summarize the latest progress in a timely manner and provide a reference for further developments with rare earth luminescent materials. This special issue includes a series of cutting-edge, high-quality original research reports and timely authoritative reviews of relevant progress involving mainly lanthanide ion-doped phosphors, persistent luminescent materials, lanthanide ion-doped upconversion materials, rare earth luminescent complexes, rare earth-relevant perovskite materials, and their applications in lighting, displays, information storage, sensing, and bioimaging/therapy.

For light-emitting diode (LED) applications, rare earth luminescent materials with high photoluminescence quantum yields, good stabilities, and broad luminescence spectra ranging from the visible (Vis) to the NIR region are highly desired. Shortwave infrared (SWIR) emission was realized by Zhang and coworkers when they doped Cr^3+^-Yb^3+^ ion pairs into Lu_0.2_Sc_0.8_BO_3_ host materials^15^. The SWIR phosphor Lu_0.2_Sc_0.8_BO_3_:Cr^3+^, Yb^3+^ exhibited high luminescence efficiency and good thermal stability, which endowed the fabricated SWIR LEDs with good performance characteristics. They further explored applications in nondestructive inspections, chemical analyses, anticounterfeiting and night vision lighting. Jin and coworkers discovered that a combination of self-trapped exciton (STE) recombination of Cs_2_AgInCl_6_ with Ln^3+^ dopant-induced extra light-emitting channels resulted in Vis-NIR (400–2000 nm) ultrabroadband emission from lead-free halide double perovskites (DPs)^16^. The fabricated LEDs featured cost-effective fabrication, compactness, excellent optical performance and extreme long-term stability compared to reported ultrabroadband light sources and showed promise for application in nondestructive spectroscopic analyses and multifunctional lighting. In addition to traditional LEDs driven by direct current (DC), alternating current light-emitting diodes (AC-LEDs) have aroused considerable interest because they can offer a more compact volume, lower production price, higher energy utilization efficiency and a longer service lifetime. To achieve white light emission during dimming time, Yuan and coworkers developed a promising strategy for building AC-LED lamps by adding a blue-light excited blue persistent luminescence (PersL) phosphor to the commercial orange‒red PersL phosphor^17^. Intense white PersL emission was observed during dimming time, with a substantial reduction in the flicker percentage.

High sensitivity, convenient use, rapid responses, and remote and noncontact properties are features of optical thermometers based on temperature-dependent spectroscopic properties, which have attracted increasing attention. However, the conventional luminescent materials applied often show background fluorescence interference due to UV/Vis light excitation. Lanthanide-doped inorganic upconversion nanoparticles (UCNPs) excitable with NIR light sources are regarded as promising alternatives with which to circumvent background fluorescence and stray light. In this special issue, An and coworkers report a novel one-step approach for synthesizing hollow structured UCNPs without a template^18^. These hollow UCNPs could act as stable self-referenced ratiometric luminescent thermometers over a broad temperature range. In addition to exploring new luminescent probes, progress has been made in relevant data processing methodologies. The power of dimensional reduction (DR) in analyses of the emission spectra for lanthanide-doped nanothermometers was demonstrated by Ximendes and coworkers^19^. Higher thermal resolution was achieved, and this may constitute a paradigm shift in the field of luminescence thermometry, particularly because it was shown that the method is applicable to rare earth luminescent materials and to semiconductor nanocrystals such as Ag_2_S.

Luminescent biodetection is another important application area for rare earth luminescent materials. Typical examples are UCNPs, where Förster resonance energy transfer (FRET) is one of the popular mechanisms adopted. Kotulska and coworkers illustrated the impact of core-shell compositional architectures of UCNPs and the mode of photoexcitation on the realization of UC-FRET from UCNPs to Rose Bengal (RB)^20^. They found that short pulsed excitation and risetime measurements provided highly sensitive assays, stressing the importance of UCNP compositional architecture for donors used to improve either the steady-state luminescence intensity or kinetic responses of UC-LRET-based assays.

Benefiting from the sharp emission and theoretical 100% internal quantum efficiency, rare earth luminescent complexes have been intensively explored over the past three decades for use in the field of electroluminescence. A typical example is organic light-emitting diodes (OLEDs). Li and coworkers presented an overview of recent advances in the use of rare earth luminescent complexes in OLEDs^21^. This review introduces the luminescent characteristics of rare earth complexes and their electroluminescent mechanisms and emphatically highlights innovative applications of rare earth luminescent complexes as sensitizers in OLEDs, including sensitizing mechanisms and the development of rare earth complex-sensitized OLEDs.

As we entered the 21st century, nano research on rare earth luminescent materials began to unfold rapidly. One such field is optical information storage. A brand new optical storage medium composed of UCNPs and EuSe semiconductors was prepared by Xie and coworkers^22^, in which the emission spectrum was excitation wavelength dependent. The long lifetime upconversion emission was filtered by time-gating technology, and the possibilities for modulating the emission colors of the nanocomposites were further expanded. Based on the multicolor tunable luminescence spectrum, the nanocomposites act as optical modules to load optical information. Furthermore, the application of rare earth-doped luminescent crystals in quantum information processing has taken a big step in the past decade with the aid of electronic-nuclear hyperfine levels, for which information on the hyperfine and superhyperfine interactions, isotopic effects, inhomogeneous line shapes, and random lattice strains in a crystal is essential. Boldyrev and coworkers presented the first direct observation of well-resolved hyperfine structure with the photoluminescence spectra of LiYF_4_:Ho single crystals and anti-crossings of hyperfine levels in a magnetic field from low-temperature high-resolution Fourier spectroscopy and a special technique for luminescence detection^23^. The results highlighted the possibility of creating a magnetic-field sensor for quantum memory devices operating at low temperatures and evaluating random lattice deformations in crystals for quantum information devices.

Rare earth luminescent materials have also exhibited potential for applications in biomedicine, especially in the diagnosis and treatment of diseases, due to their unique optical properties, superior magnetism, and high X-ray absorption coefficients. For example, rare earth NIR luminescent materials and UCNPs are regarded as potential medical theranostic platforms for diagnosis and treatment of brain diseases since NIR light can penetrate the skull. One of the challenges is that most drugs and contrast agents cannot reach and accumulate in the lesions owing to protection from the blood‒brain barrier (BBB). In this special issue, Lv and coworkers explore a Nd^3+^-doped YVO_4_ nanotheranostic agent with good BBB permeability for bimodality imaging and sonodynamic therapy (SDT) of orthotopic gliomas^24^. The nanotheranostic agent was used for highly sensitive imaging with high resolution in the second optical window (NIR-II). The role of the MnO_2_ shell is twofold: on the one hand, it catalyzes the disintegration of H_2_O_2_ to release O_2_, thereby improving SDT treatment, and on the other hand, it releases Mn^2+^ for tumor microenvironment-responsive T_1_-weighted magnetic resonance imaging (MRI). To highlight the rational design and application of rare-earth-based materials in brain imaging, therapy, monitoring, and neuromodulation, Wei et al. reviewed recent advances in the use of rare-earth-based materials for theranostic studies of brain disease^25^. This review discusses the key aspects related to functional design, structural control, and performance enhancement of rare-earth-based materials, presents perspectives and challenges for clinical applications of rare-earth-based materials and indicates that there is ample room for their use in biomedical research.

Combining UCNPs with other functional materials to construct nanocomposites and achieve synergistic effects is an important strategy for strengthening and extending the applications of rare earth luminescent materials. Du and coworkers provided a comprehensive overview of diverse designs for UCNP-based nanocomposites and outlined the pros and cons of different approaches with many instructive examples involving emerging applications in bioimaging, cancer treatments, anticounterfeiting, and photocatalytic fields^26^.

In conclusion, this special issue focuses on recent progress made with rare earth luminescent materials and considers designs, preparation methods and applications in lighting, displays, sensing, optical information storage, biomedicine, and so on. We hope that this album will encourage more scientists to enter the field of rare earth luminescent materials, which have a wide range of application prospects, inspire scientists to develop new applications and accelerate the development of rare earth luminescent materials. Finally, we would like to express our appreciation to all the authors and reviewers for their excellent contributions and critical reviews.
